# Supplementary material for: DNA polymerase ε relies on a unique domain for efficient replisome assembly and strand synthesis
Source: Nat Commun. 2020 May 15;11:2437. doi: 10.1038/s41467-020-16095-x (PMC7228970; doi:10.1038/s41467-020-16095-x)
Supplement: Supplementary file 1 — Supplementary Information [file 41467_2020_16095_MOESM1_ESM.pdf]

Supplementary Information

DNA polymerase ε relies on a unique domain for efficient replisome assembly and strand synthesis

Meng et al.

Supplementary Figure 1.

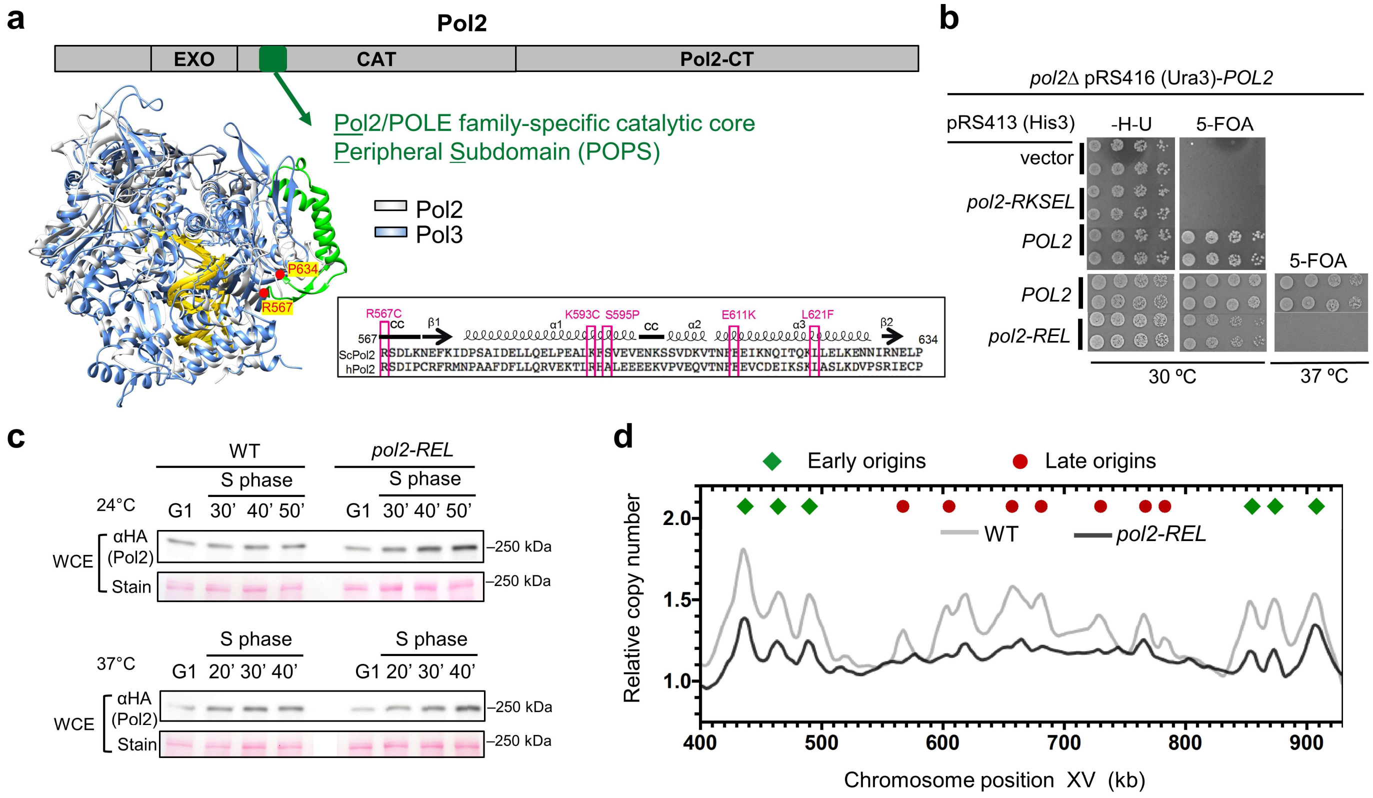

Supplementary Figure 1. Modeling cancer-associated POLE mutations at POPS in yeast reveals its contribution to DNA replication.

a. POLE mutations modeled in Pol2. Top, schematic of Pol2 domain structure. EXO: Exonuclease domain, CAT: Catalytic core, CT: C-terminal structure domain. Bottom left: the crystal structure of Pol2 N-terminal half (cornflower blue, PDB, 4M8O) is superimposed with that of the budding yeast Pol3 (light grey, PDB, 3IAY). DNA is colored gold and POPS is bright green and the amino acids marking the boundaries of POPS are labeled. Bottom right: POPS sequence alignment between Pol2 and human POLE (hPOLE) with the secondary structure of Pol2 indicated. Five cancer-associated POLE mutations are boxed purple with the residue changes in Pol2 labeled.

b. Plasmid shuffle experiments show that mutating five conserved residues in POPS leads to lethality while mutating three of these results in slower growth and temperature sensitivity. A *URA3*-marker plasmid bearing the *POL2* gene supported the viability of cells that lack the endogenous *POL2* (*pol2*Δ pRS416-*POL2*). This strain was transformed with a *HIS3*-marker plasmid (pRS413) bearing *POL2* or its mutated forms, either with five POPS mutations, *pol2-RKSEL* (*pol2-R567C, K593C, S595P, E511K, L621F*), or three POPS mutations, *pol2-REL* (*pol2-R567C, E511K, L621F*). Transformants all grew on media lacking uracil and histidine (-H-U). Forcing cells to lose pRS416-*POL2* by 5-FOA selection revealed that pRS413-*pol2-RKSEL* failed to support growth, while pRS413-*pol2-REL* permitted slower growth at 30ºC, but not at 37ºC.

c. Pol2-REL protein levels are similar to those of wild-type Pol2 in G1 and S phase cells. Experiments were done as in Fig. 1c and HA-tagged Pol2 was examined in cell extract by western blotting. Stains serve as the loading control. Similar results were obtained using two independent strains per genotype.

d. Relative DNA copy numbers in a section of chromosome XV based on genome-sequencing results. WT and *pol2-REL* cells were examined 40 min post-release at 24ºC as in Fig. 1c. Confirmed replication origins according to DNA Replication Origin Database (OriDB) [1] are labeled.

Source data are provided as a Source Data file.

Supplementary Figure 2.

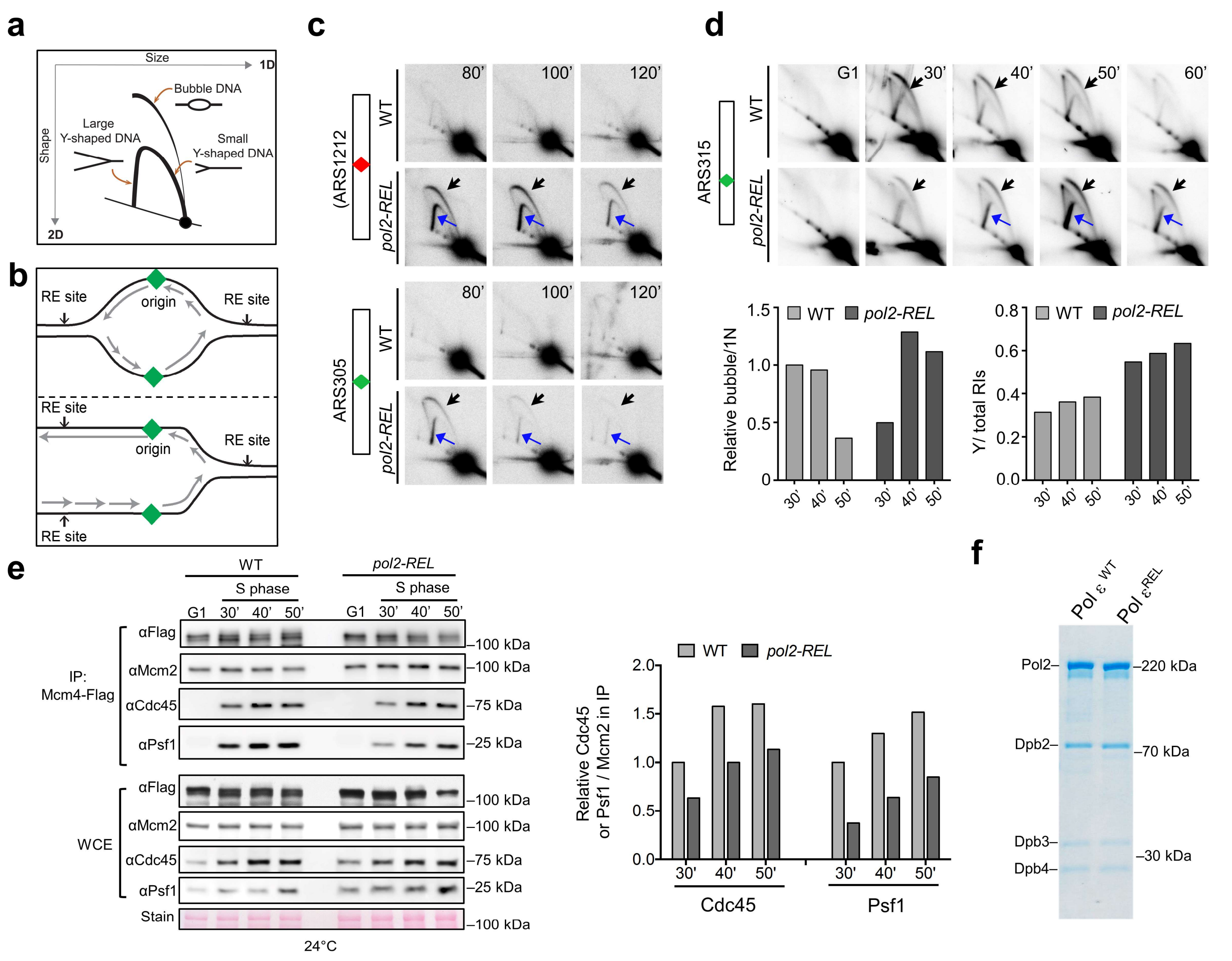

Supplementary Figure 2. Effects of *pol2-REL* on replication fork progression and CMG levels

a. Schematics to show 2D gel signals that represent different forms of replication intermediates.

b. Schematics to show replication initiation from an origin at the mid-point of a restriction fragment (RE). Symmetric replication produces bubble RIs (top), while asymmetric replication due to slowing down one of the sister replications forks give raise to Y-shaped RIs (bottom).

c. 2D gel data for later time points in the S phase show persistent replication intermediates in *pol2-REL* cells. Experiments were performed, and data are represented as in Fig. 1e.

d. 2D gel data for ARS315. Experiments were performed, and data are represented as in Fig. 1e, except that ARS315 region was examined.

e. *pol2-REL* reduces CMG levels. Experiments were performed, and data are presented as in Fig. 2a, except 24°C samples were assayed. Similar results were obtained using two independent strains per genotype.

f. Pol ε^{REL} is similarly purified as Pol ε^{WT} from yeast cells. Experimental details are described in Methods. A picture of Coomassie-stained gel is shown. Similar results were obtained from two independent experiments.

Source data are provided as a Source Data file.

Supplementary Figure 3.

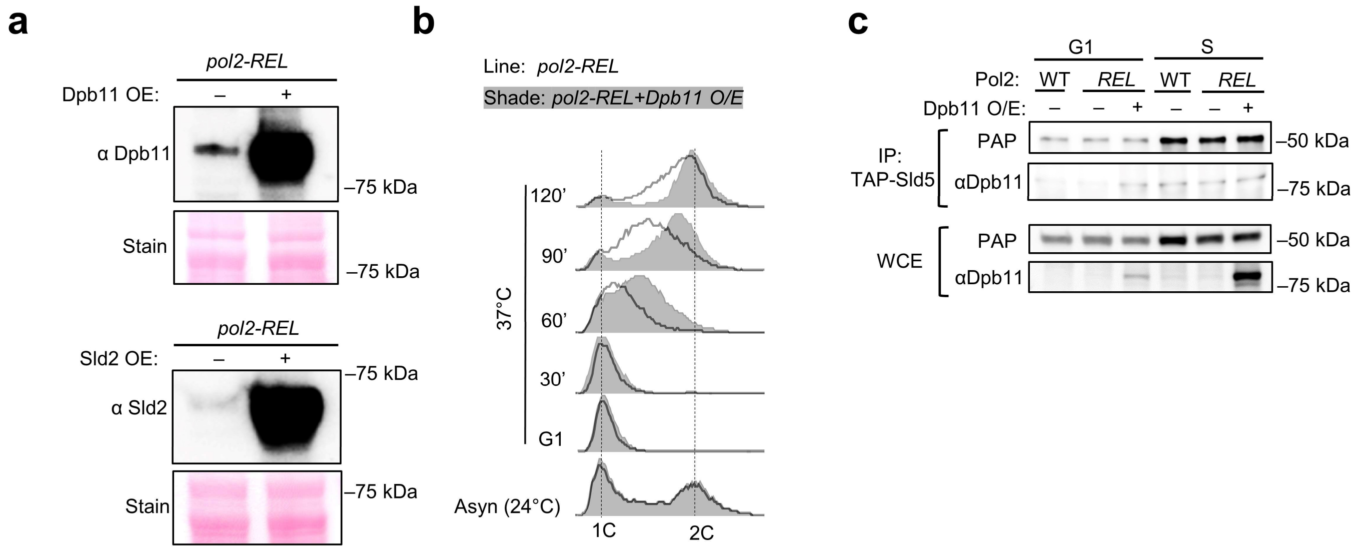

Supplementary Figure 3. The effects of increased Dpb11 dosage in *pol2-REL* cells.
a. Dpb11 and Sld2 protein levels increase upon induction. After G1 arrest at 24°C, Dpb11 or Sld2 overexpression was induced by galactose for 2 hrs. Protein extracts were examined side-by-side with endogenously expressed Dpb11 (top) or Sld2 (bottom) by Western blots.
b. Dpb11 overexpression improves replication in *pol2-REL* cells as shown by flow cytometry analyses. *pol2-REL* cells containing the Dpb11 overexpression construct (O/E, shade) or not (line) were examined as in panel (a), except that cells were further released into S phase.
c. Dpb11 overexpression does not affect its association with GINS. As in Fig. 3d, except TAP-tagged GINS subunit Sld5 was immunoprecipitated and co-purified Dpb11 was examined. For all panels, similar results were obtained using at least two independent strains per genotype.
Source data are provided as a Source Data file.

Supplementary Figure 4.

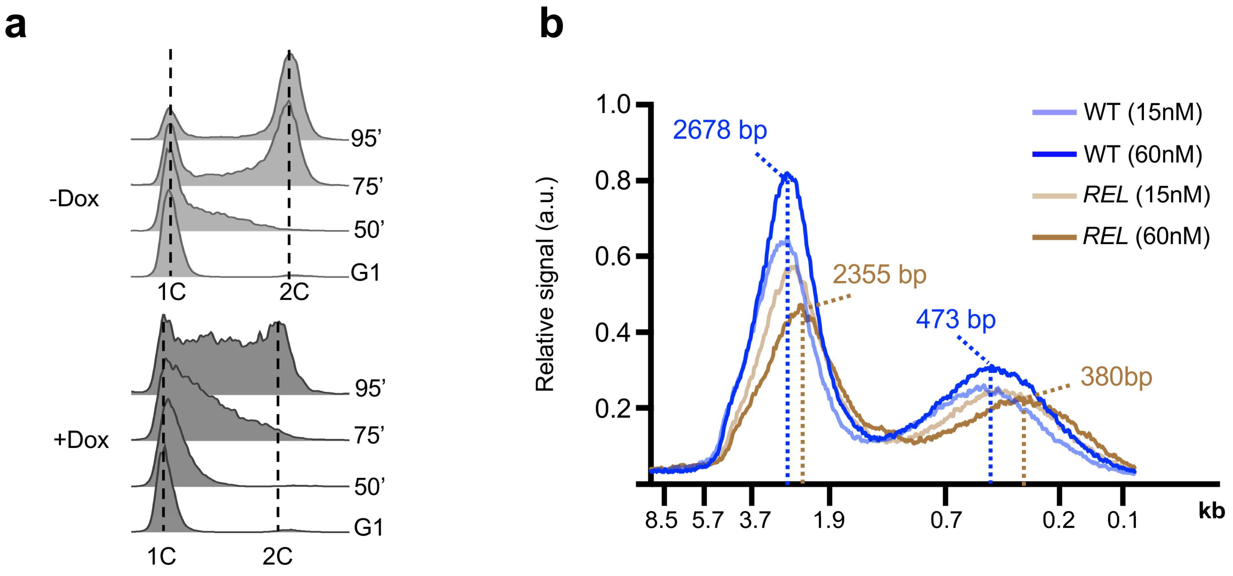

Supplementary Figure 4. Replication progression is defective in the presence of Pol ε^{REL}.

a. Flow cytometry analyses show that Dpb11 downregulation induced by doxycycline treatment slows genome synthesis compared with control cells without doxycycline treatment. Experiments were done as in Fig. 4a.

b. Traces of lanes 2, 4, 6, and 8 of Fig. 4d are plotted to illustrate the reduction in nascent leading and lagging strand lengths in the presence of Pol ε^{REL} relative to Pol ε^{WT}. The peak values of leading and lagging strand lengths are indicated.

Supplementary Figure 5.

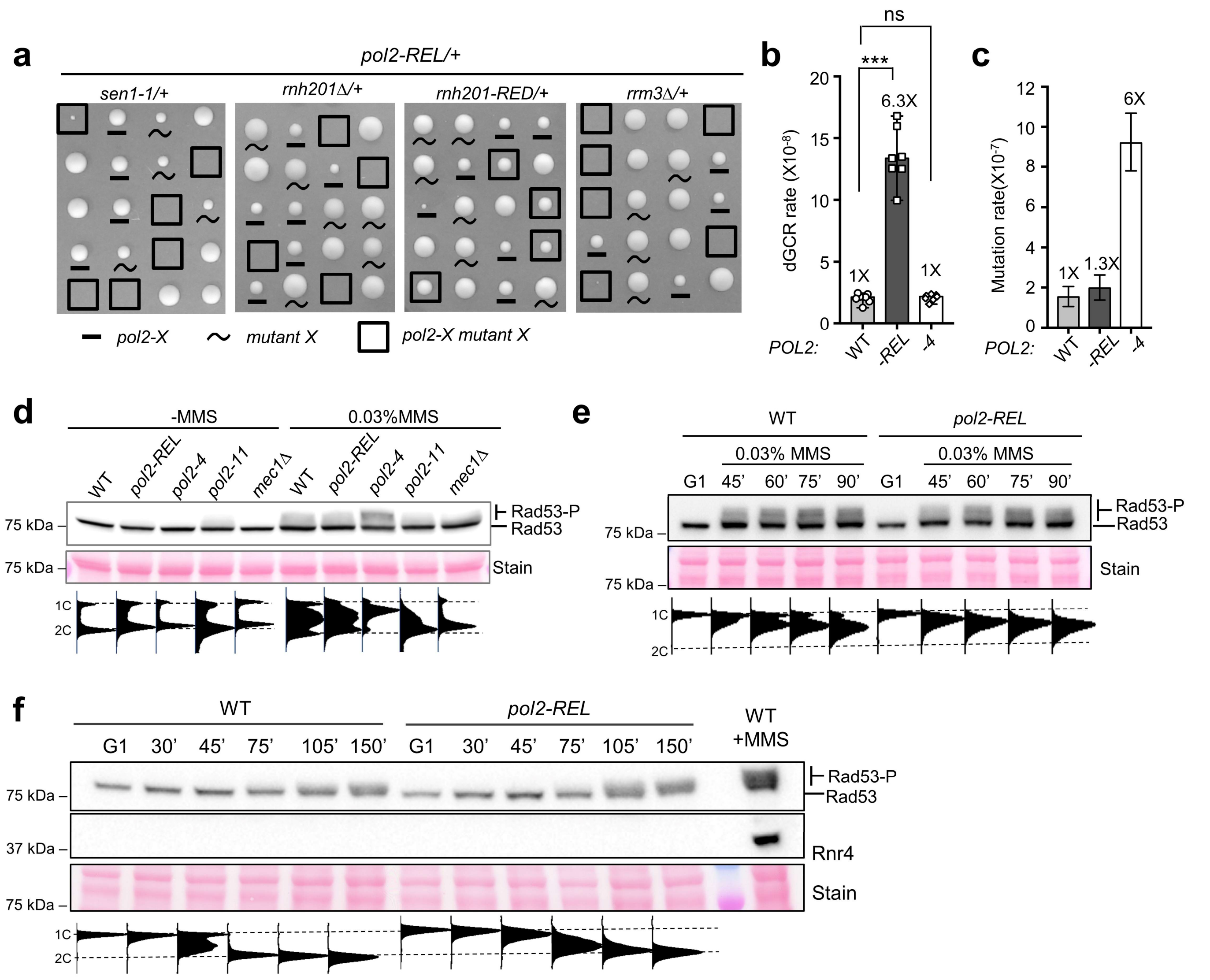

Supplementary Figure 5. *pol2-REL* is proficient for mutation avoidance and S phase checkpoint.

a. Tetrad analyses of *pol2-REL* crosses to indicated mutants. Five representative tetrads per cross are shown for each diploid with indicated genotype.

b-c. *pol2-REL* increases GCR rates but not mutation rates. Experiments were performed, and data are presented similar to Figs. 5d and 5e except that cells were examined at 24°C. Data for *pol2-4* cells are shown as a comparison. For panel b, two-tailed Mann-Whitney tests were performed for statistical analysis. ns, not significant, $p>0.05$; ***, $p<0.001$. Error bars indicate 95% confidence intervals. Data are presented as median rates of at least seven cultures from two biological replicates per genotype. For panel c, data are presented as mean rates determined from 72 cultures and two biological replicates per genotype and error bars for 95% confidence intervals.

d-e. Representative western blots to show that *pol2-REL* and *pol2-4,* but not *pol2-11,* are proficient for activating the DNA replication checkpoint upon treatment by the replication stress agent MMS. (d) Asynchronized cells of the indicated genotype were treated with 0.03% MMS for two hours. As shown previously, the upshifted band represents phosphorylated Rad53 protein, a marker for replication checkpoint. Equal loading is shown by Ponceau S staining (stain). FACS analyses (bottom) show that wild-type, *pol2-REL*, and *pol2-4* cells exhibited MMS-induced S phase arrest, whereas checkpoint defective *pol2-11* and *mec1*Δ *sml1*Δ cells failed to arrest in S phase and advanced to G2/M

phase. (e) as in (d), except that cells were first arrested in the G1 phase by alpha-factor and release into MMS-containing media for time-course analyses.

f. Time-course analyses show that wild-type and *pol2-REL* cells behave similarly in the S phase. Two checkpoint markers, including Rad53 phosphorylation and Rnr4 induction, were examined at 24ºC. Cells were collected as in Figs. 1c-1e. *pol2-REL* behaved like wild-type for examined markers during the G1 (0 min) and S phase (45 min). In G2/M phase (105 min and 150 min), *pol2-REL* cells exhibited increased Rad53 phosphorylation compared to wild-type cells, indicating the G2/M phase checkpoint activation.

For panels d-f, similar results were obtained using at least two independent strains per genotype.

Source data are provided as a Source Data file.

Supplementary Figure 6.

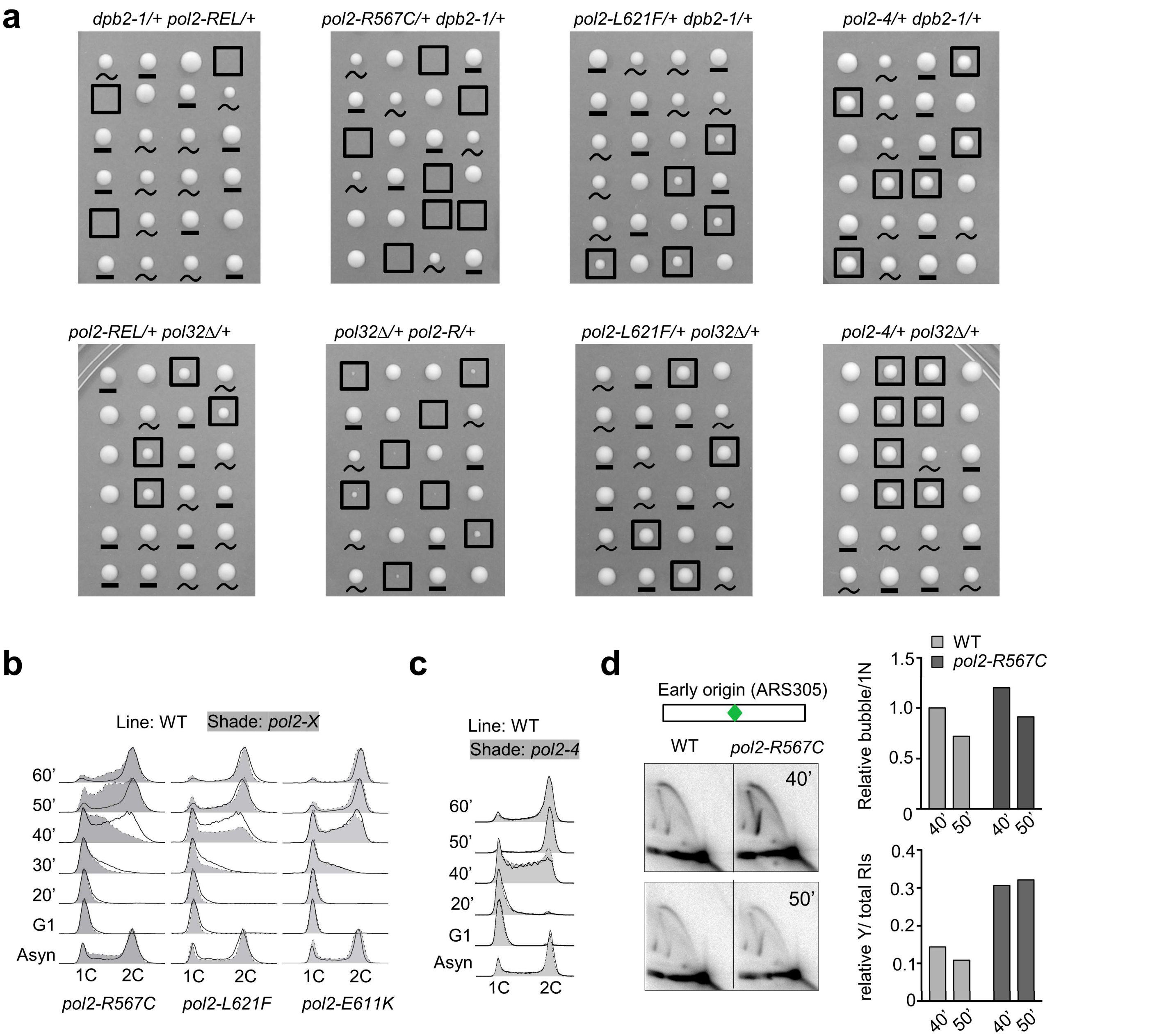

Supplementary Figure 6. Examination of single point mutations of POPS.

a. Tetrad analyses of POPS mutations and *pol2-4* crosses to either *dpb2*-1 or *pol32*Δ. Six representative tetrads per cross are shown for each diploid with indicated genotype.

b-c. *pol2-R567C* and *pol2-L621,* but not *pol2-E611K* or *pol2-4,* exhibit slow S phase progression as determined by flow cytometry analyses. Experiments were done as in Fig. 1c.

d. *pol2-R567C* cells exhibit asymmetric replication intermediates at an early origin ARS305. Experiments were conducted, and data are presented as in Fig. 6b. Similar results were obtained using two independent strains per genotype. Source data are provided as a Source Data file.

Supplementary Figure 7.

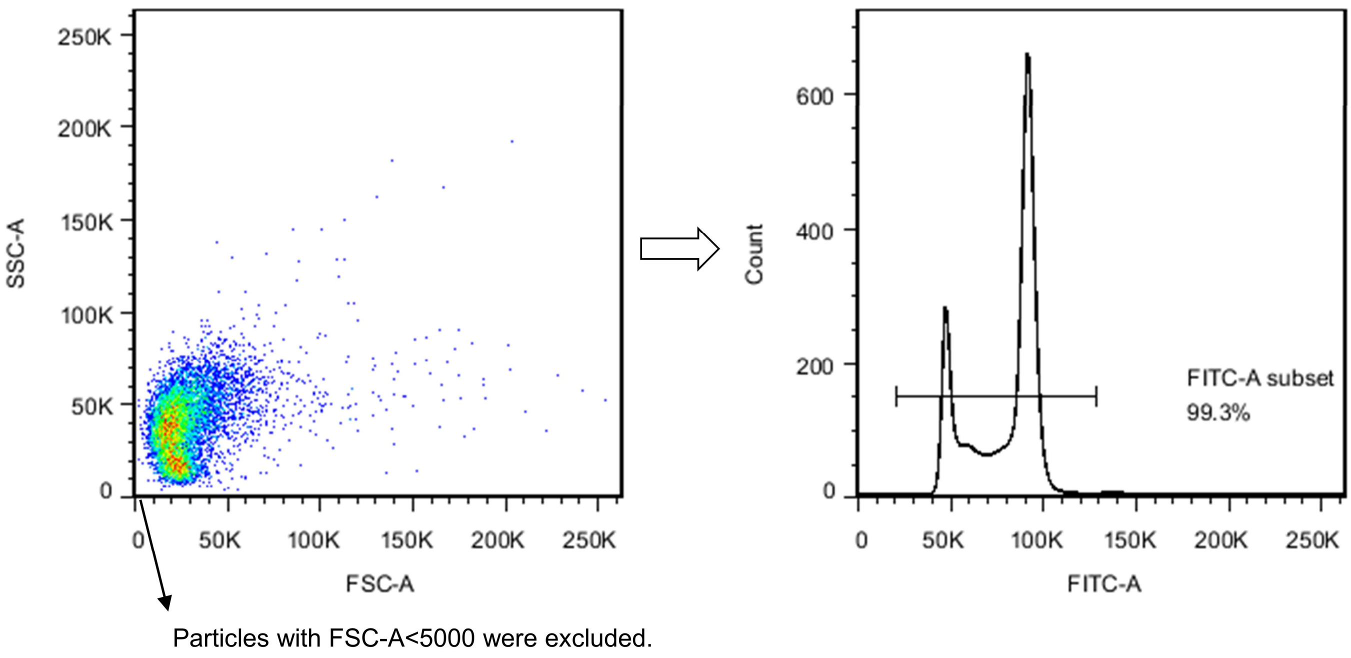

Supplementary Figure 7. Gating strategy for flow cytometry experiments.
Particles with FSC-Area smaller than 5,000 were first excluded. Gating was then performed based on the cell counts versus Sytox green intensity (FITC-A) histogram. Events with the relative FITC-A value between 20,000 and 130,000 were collected for each sample. Unstained cells were used as a negative control to determine the gate.

Supplementary Table 1. Yeast strains used in this study.

Strain	Genotype	Source
X7021-4a	*MATa POL2-3HA::KAN*	This study
X7022-7a	*MATa pol2-REL (R567C E611K L621F)-3HA::KAN*	This study
X7143-19b	*MATa POL2-3HA::KAN DPB11-9MYC::HIS3 SLD2-10FLAG::LEU2*	This study
X7144-17c	*MATa pol2-REL-3HA::KAN DPB11-9MYC::HIS3 SLD2-10FLAG::LEU2*	This study
X7091-10a	*MATa POL2-3HA::KAN MCM4-3FlAG::KAN*	This study
X7092-7b	*MATa pol2-REL-3HA::KAN MCM4-3FlAG::KAN*	This study
X7108-1c	*MATa POL2-3HA::KAN ura3::pGAL-DPB11::URA3*	This study
X7108-5c	*MATa POL2-3HA::KAN trp1::pGAL-SLD2::TRP1*	This study
X7109-2c	*MATa pol2-REL-3HA::KAN ura3::pGAL-DPB11::URA3*	This study
X7109-12c	*MATa pol2-REL-3HA::KAN trp1::pGAL-SLD2::TRP1*	This study
X7659-7d	*MATa pol2-REL-3HA::KAN ura3::pGAL-DPB11::URA3 MCM4-3FLAG::KAN*	This study
X7569-19a	*MATa POL2-3HA::KAN SLD2-10FLAG::LEU2*	This study
X7570-1c	*MATa pol2-REL-3HA::KAN SLD2-10FLAG::LEU2*	This study
X7570-18d	*MATa pol2-REL-3HA::KAN ura3::pGAL-DPB11::URA3 SLD2-10FLAG::LEU2*	This study
X7641-3a	*MATa POL2-3HA::KAN SLD2-10FLAG::LEU2 SLD5-TAP::KAN*	This study
X7642-2d	*MATa pol2-REL-3HA::KAN SLD2-10FLAG::LEU2 SLD5-TAP::KAN*	This study
X7643-10a	*MATa pol2-REL-HA::KAN SLD2-10FLAG::LEU2 ura3::pGAL-DPB11::URA3 SLD5-TAP::KAN*	This study
X7981-15c	*MATa bar1Δ POL2-3HA::KAN SLD2-10FLAG::LEU2 SSN6::pST1760(HIS3) dpb11-iAID::KAN*	This study
X8041	*pol2-REL-3HA::KAN/+ sen1-1::HIS3/+*	This study
X7990	*pol2-REL-3HA::KAN/+ rnh201Δ::KAN/+*	This study
X8269	*pol2-REL-3HA::KAN/+ rnh201-RED (P45D-Y219A)/+*	This study
X7992	*pol2-REL-3HA::KAN/+ rrm3Δ::KAN/+*	This study
X7775-4a	*POL2-3HA::KAN ura3Δ can1Δ YEL068C::URA3-CAN1*	This study
X7776-5c	*pol2-REL-3HA::KAN ura3Δ can1Δ YEL068C::URA3-CAN1*	This study
X7777-3c	*pol2-R567C-3HA::KAN ura3Δ can1Δ YEL068C::URA3-CAN1*	This study
X7772-13c	*POL2-3HA::KAN ura3Δ can1Δ YEL072W::URA3-CAN1*	This study
X7773-4c	*pol2-REL-3HA::KAN ura3Δ can1Δ YEL072W::URA3-CAN1*	This study
X7774-12a	*pol2-R567C-3HA::KAN ura3Δ can1Δ YEL072W::URA3-CAN1*	This study
X7979-1c	*pol2-4 (pol2-D290A E292A) ura3Δ can1Δ YEL072W::URA3-CAN1*	This study
X7689-3b	*MATα pol2-REL-3HA::KAN CAN1*	This study
X7690-11b	*MATα POL2-3HA::KAN CAN1*	This study
X7978-6c	*MATα pol2-4 CAN1*	This study
T2020-4	*MATa pol2-R567C-3HA::KAN*	This study
T2094-11	*MATa pol2-L621F-3HA::KAN*	This study
T2043-1	*MATa pol2-E611K*	This study
X7837-12a	*MATa pol2-4*	This study

X7505	*pol2-REL-3HA::KAN/+ dpb2-1(D300N K521R V565F G662R)/+*	This study
X7506	*pol2-R567C-3HA::KAN/+ dpb2-1/+*	This study
X7888	*pol2-L621F-3HA::KAN/+ dpb2-1/+*	This study
X7891	*pol2-4/+ dpb2-1/+*	This study
X7897	*pol2-REL-3HA::KAN/+ pol32Δ::hphNT1/+*	This study
X7907	*pol2-R567C-3HA::KAN/+ pol32Δ::hphNT1/+*	This study
X7908	*pol2-L621F-3HA::KAN/+ pol32Δ::hphNT1/+*	This study
X7909	*pol2-4/+ pol32Δ::hphNT1/+*	This study
G654	*MATa pol2-11 (Δ2192-2222)*	Previous study[2]
T1906-4	*Mat a pep4Δ::KanMX bar1::hphNAT1 Gal-Gal4 (HIS3) Gal-Dpb2/Dpb3(LEU2) Gal-pol2-REL(R567C, E611K, L621F)-CBP/Dpb4 (URA3)*	This study
YDR137	*MATa pep4::kanMX bar::hphNAT1 Gal-Gal4 (HIS3) Gal-Csm3-CBP/Tof1 (URA3)*	This study
YDR163	*MATa pep4::kanMX bar::hphNAT1 Gal-Gal4 (HIS3) Gal-Mrc1-FLAG (LEU2)*	This study
YDR110	*MATa pep4::kanMX bar::hphNAT1 Gal-Gal4 (HIS3) Gal-DPB11-CBP (URA3)*	Previous study[3]
YSD13	*MATa pep4::kanMX bar::hphNAT1 Gal-Gal4 (HIS3) Gal-Sld2-FLAG (URA3)*	Previous study[3]

Only one strain for each genotype is listed, but at least two independent isolates of each genotype were used in each of the cell-based experiments.

Supplementary Table 2. Plasmids used in this study.

Plasmid	Genotype	Source
pXZ860	*pRS416-POL2-3HA::KAN (URA3)*	This study
pXZ861	*pRS413- POL2-3HA::KAN (HIS3)*	This study
pXZ864	*pRS413-pol2-R567C, K593C, S595P, E611K, L621F-3HA::KAN (HIS3)*	This study
pXZ865	*pRS413-pol2-R567C, E611K, L621F-3HA::KAN (HIS3)*	This study
p581	*pBY011-RNH1*	Previous study[4]

Supplementary Table 3. Primers used in this study.

Experiments	Alleles/Regions	Sequences (5' to 3')
2D gel analysis	ARS305	(F) ACGCTCTGGC TTTTCGATCA
		(R) CGGACAGATTCCCCATCTCG
	ARS1212	(F) AGTTTCGGGTTCAGAGGCAG
		(R) GTCTTCACCAGCTTGGGGTT
	ARS315	(F) GTGCTGCAAAGGCCATGAAA
		(R) ACATGAACTGTATGCCCGCA
PFGE	Chr. XII	(F) TGCCGCCGACATTCTGTC
		(R) GAAACGGTGCTTTCTGGTAG
	Chr. IV	(F) TATGACCTATGGCAAACTTGG
		(R) TCTTGAACTGCGCTATCATCC
Tetrad analysis	*pol2-4*	(F) GACTTCTACTTTTTAGATGAAG
		(R) TAAATAAGAATCACGTCTCACCCAACGGAAACA
	dpb2-1	(F) GCGACGCTTCTGTCAAGATT
		(R) TTGAATGGGAATTCCAAACG
	rnh201-RED	(F) CCCACGGTAGAAGCATCCTT
		(R) AGTGATGCACGTAGTTGCGT
	rnh201Δ	(F) GGTATGCCTCCAGTAGGTGG
		(R) CAGGAAACGTAACAGCCTCTC
	rrm3Δ	(F) CACCGCTAACACAGACCC
		(R) AGGATCTGATTTCCCTCAC

Supplementary References

1 Siow, C. C., Nieduszynska, S. R., Muller, C. A. & Nieduszynski, C. A. OriDB, the DNA replication origin database updated and extended. *Nucleic Acids Res.* **40**, D682-686 (2012).

2 Dua, R., Levy, D. L. & Campbell, J. L. Role of the putative zinc finger domain of *Saccharomyces cerevisiae* DNA polymerase epsilon in DNA replication and the S/M checkpoint pathway. *J. Biol. Chem.* **273**, 30046-30055 (1998).

3 Devbhandari, S., Jiang, J., Kumar, C., Whitehouse, I. & Remus, D. Chromatin constrains the initiation and elongation of DNA replication. *Mol. Cell* **65**, 131-141 (2017).

4 Makharashvili, N. *et al.* Sae2/CtIP prevents R-loop accumulation in eukaryotic cells. *Elife* **7**, e42733 (2018).
