# Supplementary material for: DNA polymerase ε relies on a unique domain for efficient replisome assembly and strand synthesis
Source: Nat Commun. 2020 May 15;11:2437. doi: 10.1038/s41467-020-16095-x (PMC7228970; doi:10.1038/s41467-020-16095-x)
Supplement: Supplementary file 3 — Reporting Summary [file 41467_2020_16095_MOESM3_ESM.pdf]

nature research

Corresponding author(s): Xiaolan Zhao

Last updated by author(s): Apr 3, 2020

Reporting Summary

Nature Research wishes to improve the reproducibility of the work that we publish. This form provides structure for consistency and transparency in reporting. For further information on Nature Research policies, see Authors & Referees and the Editorial Policy Checklist.

Statistics

For all statistical analyses, confirm that the following items are present in the figure legend, table legend, main text, or Methods section.

n/a	Confirmed	
☐	☒	The exact sample size (*n*) for each experimental group/condition, given as a discrete number and unit of measurement
☐	☒	A statement on whether measurements were taken from distinct samples or whether the same sample was measured repeatedly
☐	☒	The statistical test(s) used AND whether they are one- or two-sided<br>*Only common tests should be described solely by name; describe more complex techniques in the Methods section.*
☒	☐	A description of all covariates tested
☒	☐	A description of any assumptions or corrections, such as tests of normality and adjustment for multiple comparisons
☐	☒	A full description of the statistical parameters including central tendency (e.g. means) or other basic estimates (e.g. regression coefficient) AND variation (e.g. standard deviation) or associated estimates of uncertainty (e.g. confidence intervals)
☐	☒	For null hypothesis testing, the test statistic (e.g. *F*, *t*, *r*) with confidence intervals, effect sizes, degrees of freedom and *P* value noted<br>*Give P values as exact values whenever suitable.*
☒	☐	For Bayesian analysis, information on the choice of priors and Markov chain Monte Carlo settings
☒	☐	For hierarchical and complex designs, identification of the appropriate level for tests and full reporting of outcomes
☒	☐	Estimates of effect sizes (e.g. Cohen's *d*, Pearson's *r*), indicating how they were calculated

Our web collection on statistics for biologists contains articles on many of the points above.

Software and code

Policy information about availability of computer code

Data collection	Flow cytometry: BD FACS Diva v6.
Data analysis	Whole Genome sequencing: FastQC v0.10.1, Cutadapt v1.9.1, BWA v0.7.8, Picard v1.110, Python v2.6, R v3.2.1.<br>Flow cytometry: FlowJo v10.<br>Statistics: Excel 2016, GraphPad Prism 7.

For manuscripts utilizing custom algorithms or software that are central to the research but not yet described in published literature, software must be made available to editors/reviewers. We strongly encourage code deposition in a community repository (e.g. GitHub). See the Nature Research guidelines for submitting code & software for further information.

Data

Policy information about availability of data

All manuscripts must include a data availability statement. This statement should provide the following information, where applicable:

- Accession codes, unique identifiers, or web links for publicly available datasets
- A list of figures that have associated raw data
- A description of any restrictions on data availability

The whole genome sequencing data associated with this study are available through GEO accession GSE132450. All other relevant data are provided in the "source data file".

Field-specific reporting

Please select the one below that is the best fit for your research. If you are not sure, read the appropriate sections before making your selection.

☒ Life sciences ☐ Behavioural & social sciences ☐ Ecological, evolutionary & environmental sciences

For a reference copy of the document with all sections, see nature.com/documents/nr-reporting-summary-flat.pdf

Life sciences study design

All studies must disclose on these points even when the disclosure is negative.

Sample size	For GCR assay and mutation rate assay, sample sizes were chosen based previous studies.<br>GCR assay: Putnam, C.D., and Kolodner, R.D. (2010). Determination of gross chromosomal rearrangement rates. Cold Spring Harb Protoc 2010, pdb.prot5492.<br>Mutation rate assay: Lang, G.I., and Murray, A.W. (2008). Estimating the per-base-pair mutation rate in the yeast Saccharomyces cerevisiae. Genetics 178, 67-82.<br>For all other experiments, each data set includes at least two biological replicates.
Data exclusions	We did not exclude data.
Replication	All the experimental findings have been reproduced in at least two biological replicates. All attempts at replication were successful.
Randomization	Samples were allocated randomly into experimental groups.
Blinding	For whole genome sequencing, GCR assay and mutation rate assay, we were blinded to group allocation during data collection and analysis.<br>For all other experiments, the blinding was not necessary and we did enough repeated experiments to get the significant results.

Reporting for specific materials, systems and methods

We require information from authors about some types of materials, experimental systems and methods used in many studies. Here, indicate whether each material, system or method listed is relevant to your study. If you are not sure if a list item applies to your research, read the appropriate section before selecting a response.

Materials & experimental systems

n/a	Involved in the study	
☐	☒	Antibodies
☒	☐	Eukaryotic cell lines
☒	☐	Palaeontology
☒	☐	Animals and other organisms
☒	☐	Human research participants
☒	☐	Clinical data

Methods

n/a	Involved in the study	
☒	☐	ChIP-seq
☐	☒	Flow cytometry
☒	☐	MRI-based neuroimaging

Antibodies

Antibodies used	anti-Dpb11 and anti-Cdc45 (from B. Stillman),<br>anti-Psf1 (from K. Labib),<br>anti-Dpb2 (from H. Araki),<br>anti-Mcm2 (Santa Cruz, sc-6680),<br>anti-Rad53 (Abcam, ab104232),<br>anti-Flag (Sigma-Aldrich, F1804, clone M2),<br>anti-Myc (Bio X Cell, BE0238, clone 9E10),<br>anti-HA (Roche, 11867423001, clone 3F10),<br>anti-Rnr4 (Abcam, ab6160, clone YL1/2).
Validation	anti-Cdc45: Sheu YJ & Stillman B et al. Cdc7-Dbf4 Phosphorylates MCM Proteins via a Docking Site-Mediated Mechanism to Promote S Phase Progression. Mol Cell. (2006)<br>anti-Psf1: Gambus A et al. GINS maintains association of Cdc45 with MCM in replisome progression complexes at eukaryotic DNA replication forks. Nat Cell Biol. (2006)<br>anti-Dpb2 and anti-Dpb11: Muramatsu S eat al. CDK-dependent complex formation between replication proteins Dpb11, Sld2, Pol ε, and GINS in budding yeast. Genes Dev. (2010)<br>anti-Mcm2: Mehanna A & Diffley J. Pre-replicative complex assembly with purified proteins. Methods. (2012)<br>anti-Rad53: Gan G et al. Helicase Subunit Cdc45 Targets the Checkpoint Kinase Rad53 to Both Replication Initiation and Elongation Complexes after Fork Stalling. Mol Cell. (2019)<br>anti-Flag: The ANTI-FLAG M2 mouse, affinity purified monoclonal antibody binds to fusion proteins containing a FLAG peptide

sequence.The antibody recognizes the FLAG peptide sequence at the N-terminus, Met-N-terminus,C-terminus, and internal sites of the fusion protein.
anti-Myc: The 9E10 monoclonal antibody reacts with human c-myc, a 62 kDa transcription factor that plays a role in cell cycle progression, apoptosis and cellular transformation. c-Myc is commonly added to proteins of interest using recombinant DNA technology.
anti-HA: Anti-HA High Affinity is a monoclonal antibody to the HA-peptide (clone 3F10). Anti-HA High Affinity recognizes the HA peptide sequence (YPYDVPDYA), derived from the influenza hemagglutinin protein. The antibody recognizes its antigenic determinant even when the HA peptide epitope is introduced into unrelated recombinant proteins by a technique known as "epitope tagging".
anti-Rnr4: Tsaponina O et al. Ixr1 Is Required for the Expression of the Ribonucleotide Reductase Rnr1 and Maintenance of dNTP Pools. PloS Genet. (2011)

Flow Cytometry

Plots

Confirm that:

☒ The axis labels state the marker and fluorochrome used (e.g. CD4-FITC).

☒ The axis scales are clearly visible. Include numbers along axes only for bottom left plot of group (a 'group' is an analysis of identical markers).

☒ All plots are contour plots with outliers or pseudocolor plots.

☒ A numerical value for number of cells or percentage (with statistics) is provided.

Methodology

Sample preparation	Yeast cells were spun down from culture, and fixed in ice old 70% ethanol. Cell were washed with and resuspended in NaCtirate buffer. RNase and Proteinase K were added sequentially to digest RNA and protein. Sytox green was used to stain the cells.
Instrument	BD LSRII
Software	BD FACS Diva Software was used for data collection, and Flowjo software was used for data analysis.
Cell population abundance	A minimum of 10,000 post-staining cells were analyzed for each condition.
Gating strategy	Particles with FSC-Area smaller than 5,000 was excluded. Gating was based on the cell counts versus Sytox green intensity histogram, and 10,000 events were collected for each sample. Unstained cells were used as negative control to determine the gate.

☒ Tick this box to confirm that a figure exemplifying the gating strategy is provided in the Supplementary Information.
